# Grassroots Governance for Sustainable Environmental Planning: Examining Manila Bay as Limit Case for Participatory Policy Implementation

**DOI:** 10.1007/s00267-026-02541-x

**Published:** 2026-08-18

**Authors:** Odessa Gonzalez Benson, Nicolás Juárez

**Affiliations:** 1 University of Michigan, School of Social Work and Taubman College of Architecture and Urban Planning, Ann Arbor, MI USA; 2 University of Michigan, School of Social Work and Department of Anthropology, Ann Arbor, MI, USA

## Abstract

In governance and social policies for environmental planning, communities are often engaged for decision-making, aspiring to fulfill democratic ideals. However, community participation is usually limited to decision making; policy implementation seems outside the scope and mandate of the community, considered separate from the governance body. This study takes up a limit case whereby community is inherent to governance. Drawing from the Philippines, we posit the *barangay* as a hybrid of sorts: local grassroots community while simultaneously formalized as ‘lowest government unit’. Using critical policy discourse analysis, we examine the 2022 Manila Bay Sustainable Development Plan for how participatory implementation is discursively planned and produced. We argue that what seems discursively produced is a devolution of state responsibilities without sufficient sharing of power, resources and knowledge needed for devolution to follow empowerment as paired logic. Yet, deliberative engagement, capacity-building and technical assistance were unmistakable, opening possibilities for participatory implementation.

## Introduction

A rich body of literature has examined how engagement, participation or input of communities and publics is key to effective environmental planning and sustainability aims (Carrick et al. 2023; Gollata et al. 2021; Hassenforder et al. 2020; Mussehl et al. 2023). James Meadowcroft (2006) notes that “the enhancement of public participation in environment and development decision-making is generally understood as an *essential* characteristic of governance for sustainable development” (162, italics added). Communities are directly impacted purveyors of local knowledge and capacities, compared to government and civil society actors whose roles are externally imposed.

Indeed, much has been written about community participation in environmental planning, with normative arguments and theoretical frameworks lending insights and mapping the conceptual course of participatory processes (Carrick et al. 2023; Gollata et al. 2021; Meadowcroft 2006; Reed et al. 2018). Scholars have examined community participation in varied contexts, with illustrative case studies that depict not only successes, but also complications and how things go wrong on the ground (Mussehl et al. 2023; Prager and Nagel 2008; Newig and Fritsch 2009). There is a wide body of literature on co-production, co-management and community-based actions in relation to the environmental policy planning and implementation, particularly on disaster risk reduction and natural resource management (Lee et al. 2024; Ranzato and Moretto 2018; Fabricius and Collins 2007).

However, communities rarely have official or formalized and compensated roles and on the ground implementation tasks, in both scholarship and practices of environmental planning. Community members are usually considered and discussed as “stakeholders,” volunteers, consumers, residents or “local resource users” in various co-management, co-production arrangements, but they typically have no legal, official, formal positions nor of payment or financial compensation (Carlsson and Berkes 2005; Lee et al. 2024). When they do have official titles or roles, community members are usually “committee members” or “board members” with advisory or consultative roles, but are often lacking in legitimacy and managerial authority, and/or not designated for positions with actual and sustained on-the-ground responsibilities and tasks for implementing policies. “Co-production is defined as voluntary efforts by individual citizens, placing it largely within the realm of charity and “third sector” work” (Watson 2014, p.65). Indeed, a critical lens on co-production has examined issues of politics, power, inequities, devolved responsibilities and resource sharing (Lowndes and Sullivan 2008; Pill 2022; Turnhout et al. 2020; Rosen and Painter 2019; Watson 2014).

When it comes to implementation especially as a particular aspect of environmental policy-practice, it is perhaps a given that implementation is beyond the role of the community, and outside its scope and mandate. Implementation “is typically done by government sector actors” (Jager et al. 2020). Local environmental policy implementation is a question of which tier of government is sufficiently local, and managing the relations and tensions in multi-tier governance (Koelman et al. 2022). The underlying logic is that the governance body is external to, separate from, and perhaps above, the community. The institutional and philosophical distance between the governing body and the community may be at the root of the assumed disassociation of community with implementation. Yet, we argue that there is room to problematize this assumption. What if community was an official part of governance, and implementation specifically? If community is at once both community as we know it, and also institutionally integral to governance, what of participation then?

This study takes up a limit case whereby community is an inherent part of *formal* governance for environmental planning. Drawing from the Philippine context, we posit the *barangay* as a hybrid of sorts: a local grassroots community that is simultaneously a political unit institutionalized as a formal, paid part of the governance body. Officially termed the Philippines’ “lowest governmental unit” whose roles were codified in the 1990s, the barangay is envisioned as site for “participatory governance” (Alip 1975; Atienza 2006; Brilliantes 1998) so that “people have more chances to participate in community activities, projects and governance itself” (Tapales n.d.). While in the U.K., the nearest analog would be the township, in the U.S. context, the village, borough or neighborhood would be the closest analog to the barangay.

In this study, we ask, how might we characterize or understand the local community’s participation via the barangay not only in deliberations and decision-making, but also in implementing an environmental sustainability plan. Using critical policy discourse analysis, we examine the 2022 Manila Bay Sustainable Development Plan in the Philippines to identify the discursive ways through which participatory implementation is planned for environmental management and sustainability.

## Background

### Environmental Planning in Manila Bay

In a 2020 *New York Times* special feature on global climate change, the Philippines’ Manila Bay was taken to be the exemplary case for rising sea waters, along with the Bay Area in San Francisco, California. “How (Manila Bay) deals with their circumstances today may offer lessons, for better or worse, for coastal cities elsewhere” (Sengupta 2020).

The urgency and gravity of these issues are not lost on local residents. In 1999, Manila Bay residents filed a legal case against several government agencies, arguing that they have neglected their duty to protect the environment and ensure communities’ safety and wellbeing. In the Regional Trial Court case, the complaint of ‘Concerned Residents of Manila Bay,’ as petitioners, stated: “[The] reckless, wholesale, accumulated and ongoing acts of omission or commission [of the defendants] resulting in the clear and present danger to public health and in the depletion and contamination of the marine life of Manila Bay, [for which reason] all defendants must be held jointly and/or solidarily liable and be collectively ordered to clean up Manila Bay…” (Concerned Residents of Manila Bay [Residents] vs Metro Manila Development Authority [MMDA], et al. 2008, p.321). Residents demanded that government agencies must “clean Manila Bay and submit[] a concerted concrete plan of action” (Residents vs MMDA 2008, p.322). And in 2008, the residents won. The Philippines’ Supreme Court mandated government action for environmental cleanup and protection, an unprecedented such case: “these government agencies are enjoined, as a matter of statutory obligation, to perform certain functions relating directly or indirectly to the cleanup, rehabilitation, protection, and preservation of the Manila Bay” (Residents vs MMDA 2008, p.328). Accordingly, the Philippines’ Department of Environment and Natural Resources assembled a team that over the next years worked on the Manila Bay Sustainable Development Master Plan, finalized in 2022.

### Community Participation and Engagement in Sustainability

In Manila Bay’s case, the community instigated governmental action. As noted above, the community’s legal action ultimately compelled the government to enact policy and plans to protect Manila Bay. This kind of deliberate engagement by the community constitutes one of the core aspects of the Plan’s approach—something we will elaborate upon below. Sustainability in environmental planning hinges on engagement precisely because ecological and social processes can be initiated and maintained only insofar as the impacted communities are involved and invested in them. Unless environmental planning is self-sustaining, it risks deteriorating without constant outside upkeep by the government.

Contemporary policy literature robustly supports this perspective. A wide range of empirical and theoretical research demonstrates that a key indicator of policy success is citizen and community involvement in decision-making regarding environmental issues and sustainability (Antunes et al. 2006; Carrick et al. 2023; De Vente et al. 2016; Jonsson et al. 2011; Mussehl et al. 2023; Prager and Nagel 2008; Reed et al. 2018). Broadly speaking, this literature identifies the need for inclusivity, community empowerment, and reflective dialogue for participatory decision-making to be productive (Carrick et al. 2023; Newig and Fritsch 2009; Reed et al. 2018).

However, while participatory engagement as an area of the policy process in decision-making is well-studied, participatory implementation is under-explored. The “link between participation and implementation may not be taken for granted” in environmental governance and there is much to be known (Jager et al. 2020). Given that community engagement and cooperation are necessary for environmental policy to succeed, examining the implementation stage of the policy process is critically important (Gibbs and Jones 2000; Mussehl et al. 2023; Reed 2008). Previous research on implementation clearly indicates that without a process to effectively channel community input and action towards sustainability, community participation tends to break down, hampering a culture of participation (Carrick et al. 2023; Reed 2008; Reed et al. 2018). As community engagement becomes an essential part of responding to the climate crisis, policymakers will need robust participatory implementation frameworks to ensure durable and effective policy responses.

### Sustainable Development and Issues of Capitalism and Power

Critical scholarship reveals that discourses and policies of development are embedded within processes of capital accumulation. Development, including sustainable development, is often mired in networks of power and social inequality (see Cervantes 2013; Escobar 2011; Raco 2005; Wanner 2015). This scholarship problematizes the assumption that development indicates progress. These critiques include examples demonstrating how development in the Global North is often linked with underdevelopment in the Global South (Murray 2008; Nef and Robles 2000; Shaikh 2005) or revealing how some development schemas reinforce and exacerbate social inequality rather than alleviating it (Peet 2010; Raco 2005).

Participation, in contrast, is frequently idealized and deployed as a response to neoliberal policies and market fundamentalism (Leal 2007; Raco 2005). *Sustainable* development sought to split, or perhaps salvage, development from its economic origins, reconfiguring it to “put greater value on environmental resources, extend[] the time horizons in which actors think and operate, and promote[] greater equity between different social groups and communities, primarily through new forms of democratic economic governance” (Raco 2005). In this way, community participation is meant to put a greater emphasis on “democratic empowerment, environmental conservation, and social justice” in contrast to neoliberal policies (Raco 2005).

### Contradictions of ‘Community Participation’: Third-order Devolution

Yet, scholars have problematized this sustaining and localizing of development unto community, when set against a capitalist, neoliberal context of power. A critical lens on community participation conceptualizes it as devolution of state responsibilities unto civil society and the private sphere, rather than a promise of democracy and empowerment (Elinoff 2021; 2014; author citation withheld). This devolution or lowering of state responsibilities—as discourses of community participation—is an enactment of the ethos of individual responsibility without structural considerations (author citation withheld; Elinoff 2021; 2014). Devolution is multi-level, devolving not only from central government to local government, conventionally examined as first order devolution; instead devolution goes ‘lower’ still (author citation withheld). Second order devolution entails subsequent transfer from local government unto civil society or the nonprofit sector; while third order devolution entails even ‘lower’ transfer from the nonprofit sector unto grassroots level (author citation withheld).

Critical scholars of social welfare have examined how “empowerment talk” and “responsibility talk” are co-constitutive in activating citizens to “participate more in society” by volunteerism and nonreliance on social services (Verhoeven and Tonkens 2013, p. 415; Newman and Tonkens 2011). More generally, devolution has long been problematized in social welfare and social development (Fording et al. 2007; Porio 2016). Similarly, in analyzing school-reform policy in Great Britain, Wright (2012) reveals how policy rearticulated education by “shift(ing) responsibility for social problems from the state to the individual… coupled with an ideological fantasy of ‘empowerment’, which conceals the subordination of actors to these neoliberal logics” (p. 279).

Studies of development in Southeast Asia too has been mired with such contradictions (Atienza 2006; Birner 2008; Kurauchi et al. 2005; Poffenberger 2006). Eli Elinoff (2021; 2014), for instance, has shown how housing programs in Thailand illustrates tensions. As implementation responsibilities are downwardly transferred to communities, “community” becomes mobilized only as a governance tool and there remains a persistent gap between the empowerment discourses put forth and the political-economic realities faced by residents. Birner (2008) has likewise found that decentralization of irrigation management in Ghana and forestry in Indonesia often shifted responsibility to local communities. Without sufficient corresponding anti-corruption safeguards, this decentralization enabled elite capture rather than genuine empowerment of the community. Both Kurauchi et al. (2005) and Poffenberger (2006) also found that local knowledges and practices were often sidelined in favor of pre-existing government frameworks. Taken together, these studies demonstrate that absent robust mechanisms for power- and resource-sharing, devolution and decentralization often strengthen unequal power relations.

Further, scholarship has also illustrated how devolution strategies have made accountability more difficult (Ferguson and Chandrasekharan 2005; Nomura 2007), including for the case of the Philippines (Manasan 2004; Punongbayan 2018). In most of these cases, the issue lied precisely in *to who* and *how* were implementing agencies accountable. As Nomura (2007) points out, the increase of international funding for environmental projects and the fact that these were carried out by environmental non-governmental organizations muddied accountability. In other cases, devolution of responsibility for implementation has led to the creation of powerful, regional leaders within Southeast Asian nations (Bhatti and McDonald 2020). When state mechanisms and resources for surveillance and anti-corruption measures are insufficient, the immense resources invested in local levels can lead to elite capture and corruption which stymies effectiveness (Shair-Rosenfield 2016). On the specific context of the barangay, Punongbayan (2018) has found that the highly fragmented mode of governance can create costly duplication in the carrying-out of everyday tasks and Manasan (2002) examines the compatibility of fiscal responsibility and local autonomy.

Relatedly, critical scholars have problematized the “myth of community” (Cannon 2024; Agrawal and Gibson 1999), cautioning against assumptions that communities are homogeneous or inherently virtuous governance actors, and romanticizing and idealizing local, indigenous leadership or organizational entities. In the Philippine context, deeper political-economic structures—including cacique democracy (Anderson 1988), local bossism (Sidel 1999), and patron-client networks (Quimpo 2005)—shape how governance operates at the barangay level. While our study does not directly assess these dynamics as we explain in the closing discussion, they provide important context for understanding how governance takes place in the Philippines since these elements of political economy necessarily shape what actions actors take as well as their relative power within the political sphere.

### The Philippine ‘Barangay’ as Limit Case

The lack of research on participation’s role in implementing environmental policy might simply be because there are so few cases to study. Implementation is a full-time job, and may thus be beyond the scope of what can be expected of or assigned to community members. While some aspects of implementation may be delegated to or shared with members of the community, communities are not commonly tasked with implementation as a whole—although we should not necessarily foreclose the idea.

The barangay presents a limit case. By limit case, it is that we consider the barangay as a boundary object of sorts, allowing interpretive flexibility, whereby it sits liminally in between two analytical categories (Hoppe 2010), in this case community and implementor or governance actor. A limit case in this sense thus does not necessarily imply that it is unique; indeed, there are similar such institutional entities, including those in Southeast Asia (i.e. Tambon Administrative Organizations in Thailand as examined by Mutebi 2004).

In the Philippines’ context, the barangay is officially termed the lowest government unit (LGU)*, structured as separate from the city or municipal government unit^1^ During pre-colonial times, it was community led by a “datu” or chieftain (Ileto 1998). The barangay was then restructured and incorporated into colonial political systems during both the Spanish colonial period (1521–1898) and the American colonial period (1898–1946) (Atienza 2006; Anderson 1988).

In 1991, the Local Government Code codified the barangay’s functions and structure (Republic Act No. 7160; Tapales 2002). Its leadership is elected: the Barangay Captain leads the entity, and the Barangay Council takes decision making and supervisory roles (Ponce 2014). Across the Philippines the population in a given barangay varies widely, with a minimum of 2000 residents (Department of the Interior and Local Government (DILG) 2018). Barangay leaders receive monthly compensation, as well as medical and insurance benefits and civil service eligibility. They are provided a budget that they manage and use, and can receive additional compensation or allowances via local resolutions when given more responsibilities. As one might expect, rural barangays have smaller population sizes compared to urban barangays. The barangay’s fourfold mandate includes: 1) enacting ordinances for things like local safety and sanitation rules; 2) delivering basic services, such as health care and waste management; 3) developing and facilitating programs, such as those for youth and women; and 4) maintaining a community-based justice system, detailed below (Atienza 2006; Capuno 2005).

We provide two examples that elaborate the barangay’s community-governance nexus. First, the barangay provides community-based justice via community ‘watch groups’ and community dispute-resolution councils (termed Barangay Peace and Order Committees), whose membership is legally required to include a public-school teacher, a representative from an interfaith group, a senior citizen, and a young person, as well as a barangay watch group member and certain other barangay officials (DILG 2018). Second, the barangay facilitates the Local Initiative and Referendum process (Sec. 120-127, LGC), a “legal process whereby registered barangay voters may directly propose, enact or amend any ordinance… Not less than fifty (50) registered barangay voters may file a petition with the barangay council concerned proposing the adoption, enactment, repeal or amendment of a barangay ordinance” (DILG 2018, page 53).

The MBSDMP itself is clear and explicit about—and celebratory of—the barangay’s dual role as both community stakeholder and governance unit. “Ultimately, the MBSDMP is not just another government’s plan but a stakeholders’ master plan for the sustainable development of Manila Bay where the active role of the (barangay) in local development (i.e., planning process, project implementation) and local governance is crucial” (p. 53).

### Conceptual Framework – Participatory Implementation in Environmental Planning

We synthesize our conceptual framework for participatory implementation in environmental planning from two areas: (a) networked/inter-organizational implementation, and (b) participatory approaches to environmental planning. The former is not specifically about environmental planning, while the latter is not specifically about implementation—so, taken together, they formulate a fuller framework. However, this combination also offers a preliminary framework for understanding the variables relevant to successful participatory implementation. We start with the four elements of interorganizational implementation in O’Toole and Montjoy (1984), then integrate them with the four elements Meadowcroft identified in his theoretical framework for participation in environmental planning (2004). Together, they comprise eight elements that we see as essential to participatory implementation.

#### Mandate

Successful implementation requires a specific mandate of action. Mandates that are imprecise or leave too much room for subjective interpretation are likely to create problems in coordinating resources and using them efficiently. However, this variable also includes clear lines of responsibility and authority for decision-making regarding implementation. If it is unclear who has the authority to issue mandates, implementation will likely run into problems compromising the successful completion of projects or even shuttering them entirely (Cisneros 2021). The result is implementation that deviates significantly from the original agreement, delays in completion, and miscommunication across coordinating agencies.

#### Monitoring

Deeply connected to ensuring clear mandates, monitoring is necessary to ensure projects are completed in a timely manner, and that actors fulfil the mandates issued by the centers of decision-making. A lack of monitoring not only likely worsens mandate specificity, but also leaves implementation vulnerable to unexpected problems. Well-coordinated and thorough project monitoring ensures that implementing actors can effectively communicate unforeseen developments to other actors to ensure that mandates are being adequately fulfilled. Monitoring is therefore not only necessary to ensure that actors fulfill their responsibilities, but also to ensure that unforeseen delays or problems are clearly communicated, and that all coordinating actors can respond effectively to such developments. However, these forms of monitoring, in turn, require strong administrative capacities and legal structures to ensure success (Steinebach 2022). Therefore, successful monitoring also requires the development of robust governmental infrastructure.

#### Resources

It is likely that decision-makers—especially those separated from direct implementation by multiple degrees—will underestimate the material, temporal, and symbolic resources necessary to ensure successful implementation of decisions. As such, successful implementation not only allocates sufficient resources for implementation, but also builds redundancy into those resources to offset possible underestimation or unforeseen issues. Participatory implementation that does not allocate sufficient resources will likely fail to achieve the original mandate and cause further delays as the implementation awaits additional resources.

#### Interdependence

Effective implementation requires successful coordination amongst deciding and implementing actors. All things being equal, the higher the number of implementing units, the greater the chance of failure. Additionally, implementing units that operate inter-organizationally tend to experience greater delays than those operating intra-organizationally, ultimately resulting in greater failures than otherwise. Only in situations where institutions pool resources towards a common target or any number of implementing agencies can complete the mandate does inter-organizational implementation tend to increase success. However, a higher implementing authority working to facilitate positive relationships and communications between implementing actors can aid implementation (Roberts and Milman 2024; Porio 2016). This suggests that greater degrees of implementation complexity *qua* greater numbers of implementing actors ought to result in a greater focus on managing that interdependence.

#### Adequate representation

While Meadowcroft (2006) interprets this to mean a diversity of stakeholders are represented in the decision-making process, when translated to implementation, it is better understood as adequately representing all actors who play a role in any work required to maintain the project being implemented. For example, if maintaining ecologically-integrated coastal defenses requires not only governmental actors, but also the local community near the coast, local fisherman, and others involved in nearby activity, then implementation should include the other non-governmental actors. Projects that fail to include multiple points of view about possible obstacles tend not only to run into unforeseen problems, but also tend to degrade over time as horizontal, ongoing social networks fail to promote projects’ successes.

#### Deliberative engagement

Successful implementation reflects robust, deliberate discussion during the decision-making process, and aligns well with the values of both the stakeholders most directly affected by the project and those involved in the day-to-day upkeep required for its ongoing success (Porio 2016; Meadowcroft 2006; Jonsson et al. 2011). Such deliberative engagement requires the openness of implementing actors to alternative approaches to implementation, and their willingness to rethink and reconceptualize ways of handling any problems within the process. To do this, not only must implementing actors have strong and consistent communication, but they must also agree to honor actors’ potentially different—even conflicting—values.

#### Different forms of knowledge

Successful participatory implementation relies on both integrating and accepting various forms of knowledge, and consulting with different knowledge-holders (Porio 2016; Meadowcroft 2006). Rather than merely rely on entrepreneurial or scientific expertise, participatory implementation requires implementing actors to draw from the wide range of social, folk, Indigenous, or otherwise marginalized non-professional knowledges from the surrounding area, and the extant literature and expertise in the problem area. Failure to integrate these knowledges can create blind spots in implementing the project, as the project’s insufficient resources or design cannot account for elements not captured through scientific investigation or through financial accounts, leading to project failure (Neuhoff et al. 2023).

#### Societal learning

Societies may not always see the value in sustainability and environmental work or in ensuring that sustainable actions continue over time; or may not have the knowledges, skills and time for action and involvement. Therefore, participatory implementation projects are conceptualized as including the promotion of values not only amongst implementing actors, but also to promote broader social transformations whose values align with sustainability and environmentalism. Failure to do this ensures that project mandates are misapplied, or that future oversight on the project falters once the original implementing actors are no longer overseeing the project (Rist et al. 2007).

## Methods

### Analyzing the Discourse of Institutional/Policy Documents

Documents are relevant to social welfare and environmental policy scholarship, as they unveil the relationship between institutional discourses and how policies are implemented (Catalano et al. 2025; Fairclough 2000; Jacobs 2006; Leipold et al. 2019; Milán et al. 2024; Rossiter 2005; Willey-Sthapit et al. 2022). As a specific theoretical and methodological approach, critical discourse analysis (CDA) is “motivated by an ambition to unmask hidden (e.g., capitalist, right-wing) ideological agendas as drivers of political text and talk, to advance democratic stakeholder participation in decision making and to critically analyze discriminatory (e.g. racist, antisemitic) language use, especially in the public sphere or by political actors” (Leipold et al. 2019; Milán et al. 2024; Schmidt 2008). As such, CDA has immediate implications for practice, as it can illuminate how ideological commitments shape institutional action (Leotti et al. 2022) as well as how the institutional contradictions revealed by discursive analysis serve to enlist or impede policy implementation (Catalano et al. 2025).

The CDA framework understands an institution’s discourse as the set of texts it produces along with the various practices of production, dissemination, and reception related to those texts (Philips 2003). Additionally, CDA views institutions as sites where various individual and collective actors vie for control and advancement of their interests (Catalano et al. 2025). Institutions, in turn, give shape, form, and unity to those conflicts (Philips 2003). By applying a critical frame to institutional discourse that reads institutions as sites of contestation, CDA advances policy research by giving scholars insight into how unspoken assumptions and ideological commitments impact how policy is implemented. This offers a point of view that other forms of policy analysis may not capture (Willey-Sthapit et al. 2022).

Discourse analyses can reveal the assumptions and ideologies embedded in sustainability policy planning (Catalano et al. 2025; Milán et al. 2024). Our CDA entailed reading across the Manila Bay Sustainable Development Master Plan (MBSDMP) for various actors’ responsibilities and entitlements. In doing so, we read the MBSDMP as a site of discussion where multiple actors speak. By searching for commonalities and contradictions in the document, it becomes possible to garner insights appropriate to the scale of the institution. Rather than begin with a given set of theoretical assumptions, our CDA is inductive. We approached the text without a given framework, instead matching discourse after the fact with potential theoretical interpretations. This allowed for a nuanced, contextual analysis of the Philippine government, minimizing the risk of confirmation bias (Vaara et al. 2010). Inductive analysis is appropriate to the investigation’s grounded theoretical approach, especially since this area is under-researched and thus may be especially prone to building contradicting theoretical frames by examining other, better researched areas. The MBSDMP is official and comprehensive, detailing all aspects and steps and stakeholders that were considered in the planning. We thus posit it as suitable for the purposes of our study. However, we also acknowledge the limited scope of our study as it does not cover a more extensive dataset. Also, we acknowledge the limits of discourse analysis as method, in that there are no on-the-ground empirical data, such as interviews with stakeholders. Nevertheless, discourse analyses may raise questions and open new lines of inquiry, that would in turn be best examined in future studies that empirically examine implementation on the ground. We consider this present study as exploratory or entry way into further research.

#### Analysis

The object of our study was the Manila Bay Sustainable Development Master Plan (MBSDMP) and its annexes. Composed of over 1200 pages, the Master Plan discusses a wide range of issues from tourism to wastewater management to ecological resources to community impacts.. Developed over the course of several years and through consultation with a variety of civil society and governmental groups, the Plan represents a wide range of voices, actors, and agendas within a single set of documents. Analyzing the document revealed the viewed actors involved, and how the Plan both challenged and reified the contemporary institutional set-up.

We applied a hybrid thematic approach, utilizing both inductive and deductive analysis (Fereday and Muir-Cochrane 2006). This methodological approach is particularly useful when a study is both exploratory but also within a domain of study about which there are existing theoretical frameworks (Fereday and Muir-Cochrane 2006). Our study is exploratory in that the Plan has not been examined vis-à-vis the barangay and implementation specifically, and so it was appropriate and productive to lean on the data at the outset initially to narrow our research question. We started with open coding, and used insights from our open codes to then identify and draw upon a conceptual framework at the next level of coding.

Using the qualitative coding software Dedoose, we first utilized in-vivo and descriptive coding of the Master Plan by coding direct quotes and coding descriptively. At this initial stage, we had an overarching research question about participation in environmental planning, but only tentative analytical plans depending on what content and details could be gained from the text. First-level codes converged around collaborations, supports/entitlements and responsibilities in the Plan. This led to a second wave of coding that assigned first-wave codes into parent codes: stakeholders (ie. federal units, barangay, civil society, for-profit entities), supports and coordination activities (ie. training, funds, oversight, accountability) and broad areas of responsibilities or tasks (ie. eco-tourism, administrative, monitoring, outreach). Upon completing the second-wave coding and with more firm sense of analyses possible, we developed a conceptual framework to use to theoretically analyze data, about stakeholders, supports/entitlements and responsibilities. Here, we drew heavily from O’Toole and Montjoy (1984) and Meadowcroft (2006) to construct eight elements of participatory implementation detailed above. Next, we conducted a third wave of coding, identifying where and how the elements of the conceptual framework showed up in the data, thus conducting an deductive, theory-informed approach to analysis. In our final analysis, we juxtaposed our findings to critical scholarship examining neoliberalism, (sustainable) development, and policy implementation, identifying where our initial insights paralleled, contradicted and nuanced the literature.

#### Findings

Our findings home in on the eight elements of participatory implementation in environmental planning—based on the synthesized conceptual framework detailed above—to critically analyze how each element is discursively evidenced in the Plan. That is, we critically argue that policy discourse produces these elements as priorities and value-laden preferences, as environmental planning codifies. Of these elements, we find six—adequate representation and deliberative engagement (combined as “community engagement”), resources, monitoring, mandate, and interdependence— as predominant but nuanced in the Plan. Two other elements—different forms of knowledge and societal learning—were relatively absent. We detail the implications of these findings in the following sections.

### Community Engagement: Adequate Representation and Deliberative Engagement

Engaging the community featured prominently as part of both the decision-making process and implementation of the Plan. As Meadowcroft (2006) outlines, obtaining engagement from a wide range of actors implicated in the plan is vital. As a starting point for our research, the “barangay as community as implementing actor” is reflected in how the plan was developed. This is clear from the Plan’s use of focus-group discussions about needs and opinions with 54 barangay communities. For barangay communities, one of the main “hindrances in implementing projects in their area… [was] viable financing and funding mechanisms” for the “community’s initiatives” (Annex 7, p. 4). Additionally, communities identified “shortcoming[s] in strictly adhering to certain laws and programs,” recognizing that “enforcement is necessary.” Focus group discussions formed a qualitative approach to discovering community needs, demonstrating that the planners sought to incorporate community decision-making early in the process.

Beyond simply surveying community needs, the Plan’s main text emphasized the need for more stakeholders to participate in implementing it. The Plan specifically stressed that “The Project engaged with the stakeholders through various levels of participation: (a) joint information generation and sharing, (b) consultation and (c) collaboration” (p. 46). Further, the Plan’s planning framework and various components were to “harmoniz[e] with all relevant plans of barangays,” (p. 40) and that “the harmonization process shall be mutually agreed upon by all concerned barangays” (p. 64). The Plan’s statement on “Information, Education, Communication, and Advocacy” as aspect of stakeholder engagement detailed:

Various types of ‘Information, Education, and Communication’ materials shall be prepared and shared with stakeholders..…These materials will help stakeholders understand their roles, responsibilities, benefits and opportunities to the realization of the MBSDMP. Further, as the Plan discussed engagement, it sought to “ensure adequate stakeholder participation through democratic, transparent and open, neutral, and timely consultation activities that involve individuals, agencies, organization, disenfranchised groups and people to be affected by transboundary impacts” (p. 65). The Plan also specified the “provision of venue by which stakeholders are able to submit their views, opinions and suggestions on various reports of the project” (p. 65).

Our findings suggest that representation was adequate, while deliberative engagement was an intentional, key part of planning. However, our analysis of forms of knowledge (below) raises the question of whether community engagement was actualized vs. idealized/aspirational.

### Resources

While resources like technical assistance (TA) and capacity building (CB) emerged as dominant elements, they were underspecified in the Plan. Both TA and CB showed up across numerous sections of the planning document. But our findings suggest that while the Plan presented opportunities for engagement and provided resources, they were limited to broad abstractions and empty references. Regarding ecotourism, the Plan noted that it would provide “technical assistance to LGUs to formulate local tourism development plans, tourism management action plans, and beach management plans” (p. 88). Further, “capacity-building of LGUs on sustainable tourism development shall be promoted to make tourism programs of the LGUs more competitive, inclusive and sustainable” (p. 44). For managerial tasks, “technical assistance and capacity building on the management of MPAs shall be provided” to the barangays (p. 80). Similarly, for ecological support tasks, the Plan specified mandates to “build the capacities of barangays on Local Habitat Protection and Management,” for instance (p. 105).

However, the Plan under-specified CB and TA: the Plan neither specified what capacities would be built, or which technicalities it would assist barangays with, nor did it mention next steps. While capacity-building and technical assistance seemed to serve as catchphrases, such references were empty of substance. In the subsection on mandate and interdependence, we detail how this vagueness resulted in inadequately preparing the barangays to take on several of the roles delegated to them.

### Monitoring

While monitoring was evident in the Plan, it seemed to function as a stand-in for enforcement to ensure compliance. The Plan provided detailed and frequent procedures for monitoring, specifying which implementing groups would be monitored, and for what. This contrasts starkly with discussions of underspecified resources, as mentioned above.

Broadly, the Plan stated that Manila Bay’s governing body must be able to “exact compliance while [it] leverage[s] existing strengths and opportunities to all concerned agencies and LGUs” (p. 56). Also, the Manila Bay Task Force, the core administrative group charged with overseeing the implementation of the Plan, “shall be enabled to strictly enforce adherence of all LGUs,” which specified the Plan’s mission and vision (p. 40). And again, the “conduct of ‘Information, Education, and Communication’ on the ICZM Framework will be essential in advocating strict compliance” (p. 40). Such explicit statements were evident throughout the Plan, demonstrating that the Manila Bay Task Force saw strict oversight as essential to the Plan’s success. Discourses of strictness, adherence and compliance were evident not only in the broad governance issues, but also in the barangay’s specific tasks. The barangay was not merely an implementing agency monitored by the MBTF, but was also charged with ensuring compliance. In discussing the plan for improving eco-tourism, the Plan explicitly stated that the barangay will “implement, *monitor, and enforce*” the Plan (p. 26, emphasis added). Similar tasks were given to the barangay in other areas, such as aquaculture practices (p. 23), precision agriculture (p. 7), and wastewater (p. 6).

In each case, our discursive findings suggest that the Planners conceived of monitoring *qua* enforcement as an important part of implementation, to ensure that compliance with the plan is clearly planned for and well-integrated. Given that the barangay identified compliance as an issue in the focus group discussions above, it seems likely that success with this area of implementation comes from following their direction on this issue.

### Mandate and Interdependence

Mandate and interdependence, we argue, become over-tasking and over-dependence. The barangay was made responsible for the bulk of implementation tasks, from administration to tourism to ecological support. As detailed below, some of these are big, new and technical roles for local level barangay leaders, who generally do not bring the skills, knowledge or technical capacities needed for such tasks (UNDP 2009). While barangays-as-community and state actors depended upon each other in an interdependent relationship, their mandates were skewed. Barangays were assigned to on-the-ground implementation as well as a higher-level mandates such as development, enforcement and monitoring, while state actors were tasked with delegating authority and setting accountability measures. This burdening of and over-reliance on the barangay-as-community, together with a lack of resources and the ambiguous technical assistance mentioned above, puts barangays in a precarious position, an argument we develop further in the discussion section below.

For administrative roles, the barangay was given set tasks. Indeed, the barangay as lowest level of governance is perhaps taken to be the appropriate scale for implementors of on-the-ground activities. For instance, the Plan mandated “barangays’ strict enforcement” (p. 68) of various aspects of the project, from “General Effluent Standards requirements” (p. 68) and “precision agriculture and aquaculture” (p. 69) to local ecotourism (p. 88).

Similarly, the Plan also relegated ecotourism roles to the barangay. These mandates were not about merely enacting or implementing activities, but about re-creating or re-developing tourism into sustainable tourism—a very high-level technical task, indeed. To summarize its all-encompassing role in ecotourism, the barangay “shall implement, monitor and enforce (the National Tourism Development Plan) to strengthen responsible and sustainable local tourism practices,” as the Plan specified (p. 88).

For ecological support roles, the barangay was tasked with broad-based activities, such as “community-based sustainable habitat management and [monitoring the] implementation of local ordinances” (p. 93) and implementing and enforcing “good aquaculture practices” (p. 85). Importantly, many of the ecological support mandates for barangays were also specific and highly technical. For wastewater and water quality management, for instance, the Plan mandated various highly technical tasks to the barangays: “ensur[ing] that septic tanks are properly designed and maintained and [that] septage services are properly executed and accomplished” (p. 68); preventing “untreated domestic wastewater from entering the bay,” (p. 101); “stop[ping] direct discharge of untreated effluents to coastal waters and rivers” (p. 68); regulating “groundwater extraction through policy issuance and [explore] alternative sources of water” (p. 77); and improving the “drainage along major rivers and waterways” (p. 104). However, as mentioned above, barangay leaders were not trained for such technical tasks (UNDP 2009), or for other important environmental tasks critical to the Plan’s success. It is also worth considering whether such mandates are beyond the scope of barangay in the first place, as stipulated in policy and borne out by the history of their roles in governance. Barangays will be successful insofar are they have sufficient technical assistance and resources and supports available to them.

### Different Forms of Knowledge

While local knowledge was not as frequently invoked in the Plan as elements like TA, CB, or monitoring, the fact that the Planners engaged in a month-long series of focus-group discussions with 45 barangay communities suggests that they valued their input. In these discussions, the planning researchers sought “to gather thoughts, insights, and inputs from the community regarding the proposed initiatives under the plan and solicit responses on how to improve the proposed actions” (Annex 7, p. 3). By gathering community perspectives, the Planners seemed to be seeking to integrate forms of knowledge that were not merely technical, administrative, or scientific.

At the same time, our analysis found it difficult to identify precisely how they integrated this knowledge into the Plan. The Planners did not reference insights from the focus-group discussions with the barangay communities as they determined action items and priorities. One focus-group insight stated, “In general, the community aspires to having a clean Manila Bay that can and will support the current as well as the future generations. Support here means providing food for their family, a source of income, another mode of transportation, options to manage waste, and recreation and relaxation” (p. 19). Another insight states, “The communities also aspire for development and progress in their areas, in having industries and factories that create sufficient viable livelihood opportunities” (p. 20). Yet, providing food and livelihoods for families does not feature in the Plan’s priorities and action items. Similarly, although communities also identified recreation and relaxation, the Plan did not include planned improvements or beautifying Manila Bay for local residents, while ecotourism was very much prioritized as a core element to the Plan.

The barangay communities were quite direct in wanting their knowledges recognized and integrated into the Plan. Focus group discussions yielded this insight: “Indigenous knowledge and practices can contribute to achieve sustainable development objectives. Thus, they shall be considered in the implementation of MBSDMP programs, activities and projects without eroding the value of indigenous knowledge and practices” (p. 19). Yet, ‘indigenous knowledge and practice’ was not at all mentioned nor referenced anywhere else in the Plan. In this sense, we see community engagement within the Plan as discussed above, but not to the ideal degree that community knowledge is integrated with different forms of knowledge. This suggests that though community knowledge is being used to form directives, it does not necessarily guide implementation.

### Societal Learning

Finally, in our analysis, societal learning was less evident in the policy, suggesting that this element of participatory implementation was also not a high priority in the Manila Bay case. “The preparation of relevant ‘Information, Education, and Communication’ materials/products for broad public education” (p. 112) is named in the Plan, and yet little detail is provided and there is no focus on developing values that align with SD. This could be taken to mean that the Planners did not consider educating the broader public to be a core part of the implementation strategy. While other mandates were specific (as shown above), the mandate for creating and disseminating ‘Information, Education, and Communication’ is vague, suggesting that its implementation will be relatively poor compared to other aspects of the plan.

## Discussion

“Devolution and empowerment” is the title of Filipino scholar Tapales’ 2002,2 article on the codifying of the barangay as a government unit in the Philippines. The devolving of governance was twinned with empowerment. Decentralized, localized, small government leads or equates to democratizing power (Atienza 2006). Indeed, this twinning serves as underpinning logic in discourses of sustainable development from the turn of the twenty-first century to present.

In the Manila Bay Plan as limit case, with the barangay serving simultaneously as both community and governing unit, herein lies a unique opportunity for working *with* community as official implementing agent. Yet, it seems that the Plan flips this on its head, as our findings suggest. In the Plan, ‘working with’ seems to swing so much to the other side that the community turns into a worker. Our findings help illustrate. Our analysis argues that the Plan’s mandates for the barangay were plentiful, spanning day-to-day operations tasks to administrative tasks in ecotourism to highly technical tasks in ecological work. Yet, high-level tasks and supervisory roles remained with state agencies. Meanwhile, capacity-building and technical assistance as resources seemed to be underspecified, reflecting that they were low priorities. Regarding monitoring, the barangay seemed a middle-man subjected to compliance and accountability measures, while it was also tasked with monitoring community members lower in the institutional structure. Moreover, uptake of local knowledge from communities seemed weak, as the Plan did little to directly link action items and priority items with the community knowledges gained. Failure to integrate these knowledges into the plan not only suggests that there could be areas where implementation will falter due to unseen complications, but also suggests it is unlikely to advance societal learning of values aligned with sustainability (Ernst 2019). Instead, it may only further reinforce the power hierarchies existent within.

Language in policy and institutional documents can be considered as not merely functional, dutiful and procedural; they also as reflecting and constituting underlying ideologies and logics that reveal themselves with critical analysis (Toft 2010; Schmidt 2008; Shin and Park 2016). At the same time, we acknowledge here the limits of our present study as discourse analysis; it is focused on one Plan and lacks data (interviews, insights) from planners, practitioners and community members/barangay leaders. Future studies could consider our exploratory findings here as raising questions for more comprehensive empirical research.

We argue that devolution of state responsibilities is discursively produced in the Manila Bay Plan, rather than community participation in its ideal form. Critical studies of development and social welfare provide empirical and theoretical underpinnings as discussed above; and our findings support this literature and aim to extend it by application specifically to the context of sustainable development and ecology in the Southeast Asia context. We argue for devolution of state responsibilities in the Manila Bay case, insofar as tri-level devolution of responsibilities, from the state to civil society to the grassroots, seemed, as our findings suggest, without sufficient sharing of power, resources and knowledge needed for devolution to follow empowerment as paired logic (author citation withheld; Elinoff 2021, 2014; Watson 2014; Rosen and Painter 2019).

Our findings speak to, extend, and complicate this literature in several ways. First, they extend Elinoff’s (2014, 2021) analysis of Thai housing programs by showing that even when a community is formally institutionalized as a government unit (i.e. the barangay), the same gap between empowerment discourse and resource-poor implementation persists—but here, the mechanism is discursively underspecified “capacity building” rather than explicit resource denial. Second, where Birner (2008) and Kurauchi et al. (2005) identified elite capture and weak accountability in natural resource decentralization, our findings complicate that picture: the Manila Bay Plan includes detailed monitoring and compliance mechanisms, yet these flow upward (the state monitors the barangay) without reciprocal accountability, suggesting that decentralization’s failures can arise not only from weak oversight but also from one‑way enforcement that burdens local actors without empowering them. Third, our analysis complicates Poffenberger’s (2006) observation that state-led projects often sideline local knowledge. In the case of the Manila Bay Plan, community knowledge is solicited via focus groups but then not visibly integrated into action items—a more subtle form of erasure that operates through acknowledgment without uptake, merely performative and rhetorical. Together, these extensions suggest that devolution’s contradictions are not monolithic but take distinct discursive forms depending on how participation, monitoring, and knowledge are framed in policy texts.

Our discourse analysis suggests, ‘devolution-and-empowerment’ decoheres from its participatory aspirations and ideals, aligning with critical literature on participation and environmental planning and development. The Manila Bay Plan demonstrates the same neoliberal dynamics documented by Elinoff, Birner, Kurauchi, and Poffenberger in other sectors (housing, forestry, irrigation) in large-scale environmental planning in Southeast Asia, with the barangay serving as the focal point for these tensions. Our discursive findings extend and nuance this literature by illustrating third-order devolution characterized by: resources (technical assistance, capacity building) as underspecified; monitoring as enforcement, without upward accountability; and while the barangay had voice and celebrated as participant, knowledge integration remained unclear.

Without robust mechanisms for sharing power, resources, and knowledge, devolution risks reinforcing the very hierarchies it purports to democratize. That said, the Plan does include deliberative engagement and capacity‑building language—elements absent in some earlier decentralization failures—suggesting that participatory implementation remains a contested terrain rather than a closed case. Future research should examine whether and how barangay leaders and residents negotiate, resist, or repurpose these Plan mandates on the ground, and whether upward accountability mechanisms can be retrofitted to balance the one‑way enforcement we identified.

Aligning with critical literature on the myth of community as discussed above (Cannon 2024; Agrawal and Gibson 1999), the Plan’s discourse often treats the barangay as a coherent, representative actor, glossing over the internal differentiation by class, kinship, and political affiliation that characterizes Philippine localities. As John Sidel’s (1999) work on local bossism illustrates, barangay captains and other local elites frequently dominate decision-making and resource allocation. Therefore, devolving implementation responsibility to the barangay is not a neutral, technical transfer but a political action that risks reinforcing and personalizing or localizing power hierarchies.

Our findings raise questions about local dynamics of power within communities as mythologized. Future research should investigate how the dynamics of devolution and decentralization examined in this paper—underspecified resources, monitoring-as-enforcement, weak integration of local knowledge—interface with these specific social and political forces, including cacique democracy (Anderson 1988), local bossism (Sidel 1999), and patron-client networks (Quimpo 2005) and the tendency of decentralization to reinforces these localized power constellations (Porio 2016). Such research would help determine whether and how these entrenched political-economic structures shape, and are shaped by, participatory implementation frameworks in environmental planning.

Notwithstanding, we emphasize the word *sufficient* in our summative thesis statement above. That is, the sharing of power, resources and knowledge is not missing from the Plan altogether; it is just insufficiently evident. As we discussed, intent and actions towards community representation and deliberative engagement seemed not only unmistakable but celebrated in the Plan, from the focus-group discussions at the outset to recognizing the importance of harmonizing the Plan with barangays’ existing local plans. Also, capacity-building and technical assistance featured prominently in the Plan, illustrating a mindset cognizant of the needs of the barangay. But it is in follow-through and specifications where the Plan seems to falter.

## Data Availability

No datasets were generated or analysed during the current study.

## Footnotes

1 We use the terms barangay and LGU interchangeably. The term barangay is used in the narrative of the paper, but we retain the use of the term LGU in quoting the document we analyze.

2 P.D. Tapales is a Filipino scholar of public administration.
